# Vascular and autonomic recovery after resistance training with and without post-exercise static stretching: a randomized controlled crossover trial

**DOI:** 10.1007/s00421-026-06411-6

**Published:** 2026-09-03

**Authors:** Salvatore Frenda, Gianmarco Pinto, Masatoshi Nakamura, Andreas Konrad, Angelo Iovane, Marianna Bellafiore, Antonino Bianco, Ewan Thomas

**Affiliations:** 1 Sport and Exercise Sciences Research Unit, Department of Psychology, Educational Science and Human Movement, University of Palermo, Via Giovanni Pascoli 6, Palermo, 90144 Italy; 2 Faculty of Rehabilitation Sciences, Nishi Kyushu University, Kanzaki, Saga Japan; 3 Institute of Human Movement Science, Sport and Health, University of Graz, Graz, Austria

**Keywords:** Strength training, Flexibility training, Vascular health, Recovery, Arterial stiffness, Heart rate variability, Hemodynamic

## Abstract

**Purpose:**

Resistance training (RT) may induce transient changes in endothelial and autonomic patterns. Static stretching (SS) performed at the end of an RT session may alter the temporal course of recovery of these responses. This study investigates whether SS following RT modulates acute endothelial, autonomic, and haemodynamic recovery compared with RT alone.

**Methods:**

Twenty-seven healthy males completed three experimental sessions in a randomized-crossover design: RT, RT + SS, and control condition (CC). RT consisted of two upper-body resistance exercises (bench-press and seated-biceps curl), while RT + SS included the same RT protocol followed by SS of the same muscle groups. Flow-mediated dilation (FMD%), brachial-artery diameter (BA diameter), heart rate variability (HRV), heart rate (HR), and mean arterial pressure (MAP) were measured at baseline (BL), immediately after exercise (POST), and 30 min after exercise (POST30).

**Results:**

RT produced a significant reduction in FMD% at POST (*p* < 0.001; Cohen’s d = 1.58), which remained below the BL value at POST30 (*p* = 0.012; d = 0.90); these decreases were not observed in RT + SS. The BA diameter increased following both conditions; however, at POST30 it remained above BL only following RT (*p* < 0.001; d = 0.60). At POST, both conditions significantly reduced RMSSD and HF, along with an increase in HR. At POST30, however, HF and RMSSD showed an unfavorable recovery in RT alone (*p* = 0.016; d = 0.72; *p* = 0.028; d = 0.57), while in RT + SS all values returned to BL. MAP decreased at POST in both conditions and returned to BL at POST30.

**Conclusion:**

The addition of SS at the end of a RT session was associated with earlier autonomic and vascular recovery compared with RT alone. SS may represent a simple and sustainable recovery strategy after RT.

**Supplementary Information:**

The online version contains supplementary material available at https://doi.org/10.1007/s00421-026-06411-6.

## Introduction

Current Guidelines recommend resistance training (RT) to improve muscle strength, functional capacity, and overall health (McLeod et al. 2024). Although RT is a fundamental component of exercise programs aimed at improving performance and preventing numerous chronic conditions (Ciccolo et al. 2010), previous evidence suggests that acute exercise stress may transiently alter vascular and autonomic function (Dawson et al. 2013; Franklin et al. 2020), and similar responses have also been observed following particularly vigorous RT protocols (Esaki et al. 2026; Wang et al. 2024). These acute responses are relevant because they may influence the early post-exercise recovery period and may also contribute to the physiological response underlying long-term vascular adaptations (Kingsley and Figueroa 2016; Oliveira et al. 2012). Static stretching (SS) is a method generally used to improve range of motion (ROM) (Konrad et al. 2024a, b), but a growing number of studies suggest that it may also induce favorable vascular responses (Thomas et al. 2021; Warneke et al. 2025b). Recent evidence indicates that performing SS after a high-intensity strength training session may accelerate the return to baseline values of certain indices of arterial stiffness (Esaki et al. 2026). However, the physiological implications of a more rapid return to baseline values remain unclear, particularly in healthy young adults, and it is also unknown whether similar trends are observed for endothelial function and cardiac autonomic modulation. Since SS is a low-intensity intervention with limited metabolic demand, it can easily be incorporated at the end of an exercise session and represents a practical model for studying early post-exercise recovery responses.

One of the accepted non-invasive vascular parameters that provides a comprehensive picture of endothelial function by measuring arterial reactivity is flow-mediated dilation (FMD) (Ahn et al. 2024); when combined with changes in the diameter of the brachial artery, it offers complementary information useful for contextualizing vascular response to the various training stresses administered (Shivgulam et al. 2023). In addition to vascular function, heart rate variability (HRV) provides information on autonomic modulation (Shaffer and Ginsberg 2017), while mean arterial pressure and heart rate reflect the systemic hemodynamic response (Li et al. 2023). Therefore, the simultaneous assessment of endothelial, autonomic, and hemodynamic variables can offer a more integrated understanding of post-exercise recovery compared to the analysis of a single physiological outcome. Moreover, it is not yet clear whether SS performed immediately after RT can influence the acute recovery of endothelial, autonomic, and hemodynamic responses. Therefore, the aim of this study was to evaluate whether adding SS after an RT session influences acute endothelial, autonomic, and hemodynamic responses during early post-exercise recovery. These findings may contribute to a more precise characterization of the acute vascular, autonomic, and hemodynamic responses associated with the addition of SS during the early recovery phase following RT. We hypothesized that RT would induce transient reductions in endothelial function and HRV indices, along with acute hemodynamic changes, and that the addition of SS would attenuate these responses and facilitate a more rapid return to baseline values during the early recovery period.

## Materials and methods

### Participant recruitment and study design

This study was conducted as a randomized crossover trial and followed the CONSORT recommendations for crossover studies (Dwan et al. 2019) (see Supplementary Material, Online Resource 1 [ESM_1.docx]). Participant recruitment took place between November 2025 and February 2026 and involved a population of university students. A priori power analysis was performed using G*Power software for a repeated-measures ANOVA that included an intra-group/inter-group interaction. Assuming a mean effect size (f = 0.30), a significance level of α = 0.05, and a statistical power of 0.80 (Yu et al. 2025), the analysis indicated that a minimum sample size of twenty-seven participants was required. Consequently, twenty-seven healthy physically active young male university students were enrolled (25.18 ± 2.4 years; 74.17 ± 5.79 kg; 171.83 ± 5.35 cm). Eligibility criteria required that participants be free of orthopedic, neurological, renal, metabolic, and cardiovascular disorders and not be taking antihypertensive medications. Prior to participation, all individuals received a detailed explanation of the study procedures, potential risks, and expected benefits, and subsequently provided written informed consent. No adverse events were reported during the experimental sessions, and all enrolled participants completed all conditions. If serious adverse events had occurred, they would have led to the immediate termination of the trial. The study protocol was approved by the Bioethics Committee of the University of Palermo (protocol no. 353/2025) and was conducted in accordance with the Declaration of Helsinki and European Union guidelines for good clinical practice. The trial is registered on ClinicalTrials.gov (Identifier: NCT07364149).

### Randomization, allocation and blinding

All participants completed three experimental conditions: an RT condition, an RT + SS condition, and a seated control condition (CC). The order of the conditions was determined using simple randomization generated via an online randomization tool (www.randomizer.org). Randomization, participant recruitment, and data collection were managed by the same researcher. The RT condition was coded as “1”, the RT + SS condition as “2”, and the CC as “3”. Participants were assigned numbers corresponding to the randomized sequence. The assignment was kept hidden from participants until their arrival for the first experimental session. Due to the nature of the interventions, it was not possible to blind the participants, and no blinding was implemented for the researchers.

### Procedure

Each participant completed four laboratory visits scheduled for the same time of day and spaced at least 72 h apart, in order to minimize potential carryover effects. All experimental sessions were conducted at the Functional Assessment Laboratory of the University of Palermo, in a quiet, temperature-controlled environment maintained at 24 °C. Before the start of the three experimental conditions, all participants took part in a preliminary familiarization session in the laboratory. During this visit, baseline anthropometric measurements were collected, including height, body weight, body composition, and medical history. During the same session, participants were instructed on the experimental procedures and the exercises included in the training protocol. Additionally, the load corresponding to 8 repetitions maximum (8RM) was determined for the selected exercises in order to customize the intensity of the intervention during subsequent experimental visits (Simão et al. 2005).

Before each visit, participants were instructed to avoid exercise for at least 24 h beforehand, and to avoid alcohol and caffeine for at least 12 h, while maintaining their usual sleep patterns. In addition, they were asked not to eat for at least 6 h immediately prior to the assessment. During each visit, all outcomes were collected at three time points: after the initial stabilization period (baseline [BL]), immediately after completion of the assigned condition (POST), and 30 min after completion of the assigned condition (POST30). These time points were selected a priori to capture two distinct phases of the acute cardiovascular response. The POST time point was designed to capture the immediate endothelial, autonomic, and hemodynamic disturbances induced by the assigned condition, while the POST30 time point was selected to assess early recovery and determine whether these responses persisted, returned to BL values, or differed across the various conditions. The 30 min interval was consistent with previous studies on acute endurance exercise that used this time interval to assess early endothelial and autonomic recovery (Tsuchiya et al. 2023; Heffernan et al. 2006; Boeno et al. 2019; de Oliveira et al. 2020). The collected outcomes were: blood pressure (systolic [SBP], diastolic [DBP] and mean arterial pressure [MAP]), heart rate (HR), flow-mediated dilation (FMD%) and brachial artery diameter (BA diameter), and heart rate variability (HRV).

During each lab visit for either experimental or CC, each participant had to undergo an initial stabilization period of 5 min. Following the stabilization period, each participant underwent the assessments in a standardized order (BP, HRV, and FMD%), which was identical across all experimental conditions and time points. All assessments were performed by the same researcher to ensure maximum consistency and procedural reproducibility and to minimize inter-rater variability.

### Outcomes

#### Flow-mediated dilation and brachial artery diameter

FMD% and BA diameter were assessed using a MyLab X1 Go ultrasound system equipped with an LA154BH linear probe (Esaote, Genoa, Italy). Image acquisition procedures were standardized according to current guidelines for FMD assessment (Thijssen et al. 2019). FMD% was calculated as the percent change from the mean baseline diameter, averaged over the 1 min pre-occlusion period, to the maximum diameter recorded within the 3 min period following cuff deflation after 5 min of occlusion (Yamato et al. 2016).

$$ {\text{[(peak diameter}} - {\text{baseline diameter)/baseline diameter]}}. $$

Ultrasound videos were exported in MOV format and subsequently analyzed using a modified version of *FloWave.US* (Masterova et al. 2024), an open-source MATLAB-based software optimized for the automated analysis of FMD% and vessel diameter.

#### Heart rate variability

HRV was recorded using a Polar H10 chest strap (Polar Electro Oy, Kempele, Finland). Participants rested in the supine position for 5 min, after which a 5-min recording was obtained in the same position under spontaneous breathing conditions. RR interval data were acquired using the Elite HRV mobile application (Elite HRV, Asheville, NC, USA) and subsequently analysed using Kubios HRV Scientific Lite version 4.1.2.1 (Kubios Oy, Kuopio, Finland). For each assessment, a continuous 5-min segment was analysed. RR intervals identified as artefacts were corrected using the threshold-based beat correction method at the medium setting (displayed threshold: 0.30 s). Approximately 0.56% of all analyzed RR intervals were corrected. The RR interval time series were resampled at 4 Hz using cubic spline interpolation and detrended using the smoothness priors method with a smoothing parameter of λ = 500, corresponding to a cut-off frequency of 0.035 Hz. Power spectral density was estimated using a fast Fourier transform-based Welch periodogram. The low-frequency (LF) and high-frequency (HF) bands were defined as 0.04–0.15 Hz and 0.15–0.40 Hz, respectively. Time-domain indices included the standard deviation of normal-to-normal RR intervals (SDNN) and the root mean square of successive differences between normal-to-normal RR intervals (RMSSD). Frequency-domain indices included LF power, HF power, and the LF/HF ratio. LF and HF power were expressed in absolute units (ms²), whereas the LF/HF ratio was dimensionless. Mean HR was derived from the same 5-min recording.

#### Mean arterial pressure

MAP was calculated from the systolic and diastolic blood pressure values obtained using a validated automatic oscillometric device (Omron MX3 Plus; Omron Healthcare, model HEM-742-E) (Coleman et al. 2005). Before each measurement, participants remained lying supine at rest for 5 min to allow for hemodynamic stabilization. The cuff was placed on the bare arm at heart level. Three consecutive measurements were taken at each time point, and the mean value was used for analysis. MAP was then calculated using the standard formula: MAP = DBP + 1/3 (SBP − DBP), where DBP represents diastolic blood pressure and SBP represents systolic blood pressure.

### Interventions

#### Resistance training intervention

The RT intervention consisted of two upper-body strength exercises: bench press performed on the Smith machine and seated biceps curl exercises. For each exercise, participants performed 4 sets of 8 repetitions using the previously determined maximum sustainable load (8RM), under the supervision of the experimenter. The order of the exercises was randomized, and a 2-minute rest period was allowed between each set. To minimize potential bias related to differences in post-exercise recovery duration, participants in the RT condition remained seated for 12 min after the end of the last set, so that the timing of the post-intervention assessments corresponded to the duration of the SS protocol performed in the RT + SS condition.

#### Resistance training and static stretching intervention

The RT + SS intervention consisted of the same RT protocol used in the RT condition, followed by SS exercises targeting the major muscle groups involved during the resistance exercises, namely the pectoralis major and elbow flexors. Two exercises were performed bilaterally, whereas one was performed unilaterally on each side. Each SS exercise was performed for four sets of 30 s, interspersed with 30 s of rest, with the order of the exercises randomized. The total duration of the SS protocol was 12 min. The SS intensity was set at 8/10 on a subjective discomfort scale, where 0 indicated no discomfort and 10 indicated the maximum tolerable (Warneke et al. 2025a), and participants were asked to maintain this intensity, adjusting their stretching position as needed to preserve the assigned intensity. It has previously been demonstrated that individual assessments of stretching intensity are associated with passive torque and range of motion, confirming their usefulness in personalizing and standardizing the stretching stimulus (Freitas et al. 2015).

#### Control condition

During the CC, participants remained seated in a chair without performing any exercises or stretching for a period comparable to the total duration of the RT + SS condition. All outcomes were assessed at the same time points as those used in the experimental conditions: BL, POST, and POST30.

### Statistical analysis

The data are reported as means ± standard deviations. Potential baseline differences among the three experimental conditions (RT, RT + SS, and CC) were examined using a one-way analysis of variance (ANOVA). The reliability of the three baseline assessments across the experimental conditions (RT, RT + SS, and CC) was evaluated using intraclass correlation coefficients (ICC) for FMD%, BA diameter, and MAP. For HRV indices, the reliability of baseline RR interval measurements between the three experimental conditions (RT, RT + SS, and CC) was additionally evaluated using ICC. To assess condition × time interactions for all outcome variables, a repeated-measures analysis of variance (RM-ANOVA) was performed with a 3-condition × 3-time-point design. The within-subjects factors were condition (RT, RT + SS, and CC) and time (BL, POST, and POST30). The assumption of sphericity was tested using Mauchly’s test, and when violations were detected, the degrees of freedom were corrected using the Greenhouse-Geisser correction. Effect sizes for the ANOVA models were expressed as partial eta-squared (ηp²), with values between 0.01 and 0.059 considered small, between 0.06 and 0.139 moderate, and ≥ 0.14 large (Cohen 2013). When a main effect or significant interaction was observed, Bonferroni-adjusted post-hoc analyses were conducted to identify specific differences between and within conditions. In addition, Cohen’s d was calculated for pairwise comparisons and interpreted as trivial (0.00–0.19), small (0.20–0.49), moderate (0.50–0.79), or large (≥ 0.80) (Cohen 2013). All statistical analyses were performed using JASP (Version 0.96) (JASP Team 2026), with statistical significance set at *p* < 0.05.

## Results

### Comparison of baseline values of between conditions

No significant differences in baseline values were observed across the three experimental conditions (RT, RT + SS, and CC) for any of the variables assessed, including FMD%, BA diameter, HRV, MAP, HR.

### Measurement reliability across baseline assessments

The BA diameter showed good reliability, with an ICC of 0.79. For HRV, the RR intervals also demonstrated good reliability (ICC = 0.79), while MAP showed moderate reliability, with an ICC of 0.62. Lastly, the ICC for FMD% was 0.36.

### Flow-mediated dilation and brachial artery diameter changes

**Fig. 1:**
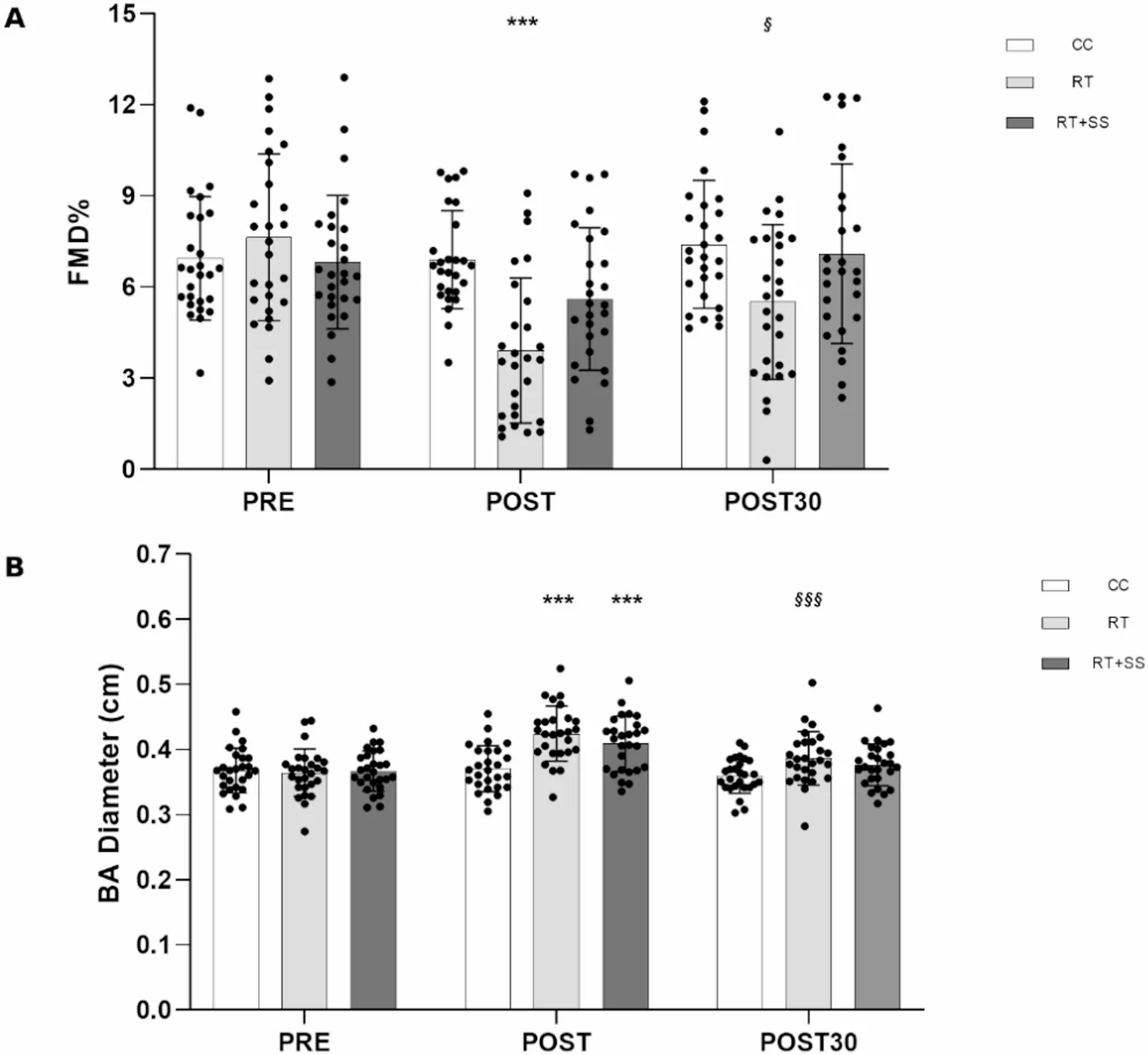
Flow-mediated dilation (**a**) and brachial artery diameter (**b**) measured at baseline (PRE), immediately after the intervention (POST), and 30 min post-intervention (POST30) during the control condition (CC), resistance training (RT), and resistance training followed by static stretching (RT + SS). Data are presented as mean ± SD with individual participant values. *p < 0.05, **p < 0.01, ***p < 0.001 for comparisons between POST and PRE within the same condition. **§**p < 0.05, **§§**p < 0.01, **§§§**p < 0.001 for comparisons between POST30 and PRE within the same condition

Significant condition × time interactions were observed for both variables (*p* < 0.001); the respective time patterns across the different conditions are shown in Fig. 1.

Bonferroni-adjusted post hoc analyses showed a significant reduction in FMD% from BL to immediately POST in the RT condition (*p* < 0.001; Cohen’s d = 1.58), and FMD% remained lower than the BL value at POST30 (*p* = 0.012; d = 0.90). In contrast, no significant reduction in FMD% was observed at any time point in the RT + SS condition. Furthermore, significant changes in BA diameter were observed in both exercise conditions. In the RT condition, the BA diameter increased from BL (*p* < 0.001; d = 1.65). A significant increase from BL was also observed in the RT+SS condition (*p* < 0.001; d = 1.16). At POST30, however, the BA diameter remained significantly elevated in the RT condition (*p* < 0.001 vs. BL; d = 0.60), while in the RT + SS condition it returned to BL values. No significant changes were observed in the CC.

### Heart rate variability changes

The RM-ANOVA outcomes for HRV parameters are summarized in Table 1. A significant condition × time interaction was observed for all variables (from *p* < 0.001 to *p* = 0.002). Post-hoc analyses with Bonferroni correction revealed a significant reduction in RR intervals from BL to immediately after (POST) in both exercise conditions (RT: *p* < 0.001; Cohen’s d = 1.59; RT + SS: *p* = 0.001; d = 1.30) and remained significantly different at POST30 (RT: *p* < 0.001; d = 0.74; RT + SS: *p* = 0.013; d = 0.58).

Regarding frequency domain indices, LF decreased significantly only in the RT + SS condition at POST (*p* = 0.004; d = 0.97), while HF decreased significantly from BL to POST in both exercise conditions (RT: *p* < 0.001; d = 1.00; RT + SS: *p* = 0.002; d = 0.85). At POST30, HF remained significantly different from BL only in the RT condition (*p* = 0.016; d = 0.72). The LF/HF ratio was significantly increased only at POST in the RT condition (*p* = 0.002; d = 1.08).

**Table 1:** Synthesis of HRV indices results for each condition and time point.

Analyzed variable	RT			RT + SS			CC			RM-ANOVA Time × condition interaction
	PRE	POST	POST30	PRE	POST	POST30	PRE	POST	POST30	
SDNN (ms)	55.11 ± 16.13	30.18 ± 15.27*	52.8 ± 8.69	57.22 ± 15.88	31.02 ± 12.54*	54.52 ± 19.29	54.88 ± 19.56	59.75 ± 23.51	63.16 ± 21.51	*p *< 0.001; F = 18.45; np^2^=0.321
LF (ms^2^)	1226 ± 731.9	565.4 ± 500.8	1023 ± 1049	1608 ± 1356	566.4 ± 382.9*	1366 ± 1149	1270 ± 843.3	1624 ± 1573	1749 ± 1412	*p *= 0.002; F = 4.55; np^2^=0.105
HF (ms^2^)	1535 ± 1284	399 ± 627*	720.3 ± 775.7*	1363 ± 908.1	399 ± 509.1*	1315 ± 1212	1687 ± 1656	1787 ± 1406	1603 ± 1275	*p *< 0.001; F = 7.64; np^2^=0.164
LF/HF (ms^2^)	1.26 ± 1.21	3.01 ± 2.45*	2.17 ± 1.82	1.78 ± 2.3	2.49 ± 1.94	1.49 ± 1.19	1.32 ± 1.06	1.08 ± 0.73	1.32 ± 0.82	*p *= 0.002; F = 4.66; np^2^=0.107

Data are expressed as mean ± standard deviations

RT: Resistance training. SS: Static stretching. CC: Control condition. PRE: Baseline Assessment. POST: Assessment after the end of the exercise session; POST30: Assessment 30 min after the end of exercise. SDNN: standard deviation of normal-to-normal intervals; LF: low-frequency power; HF: high-frequency power; LF/HF: low-frequency/high-frequency ratio. ms: milliseconds; ms²: milliseconds squared; RM-ANOVA: Repeated Measures Analysis of Variance

*Significant difference compared to baseline (PRE)

**Fig. 2:**
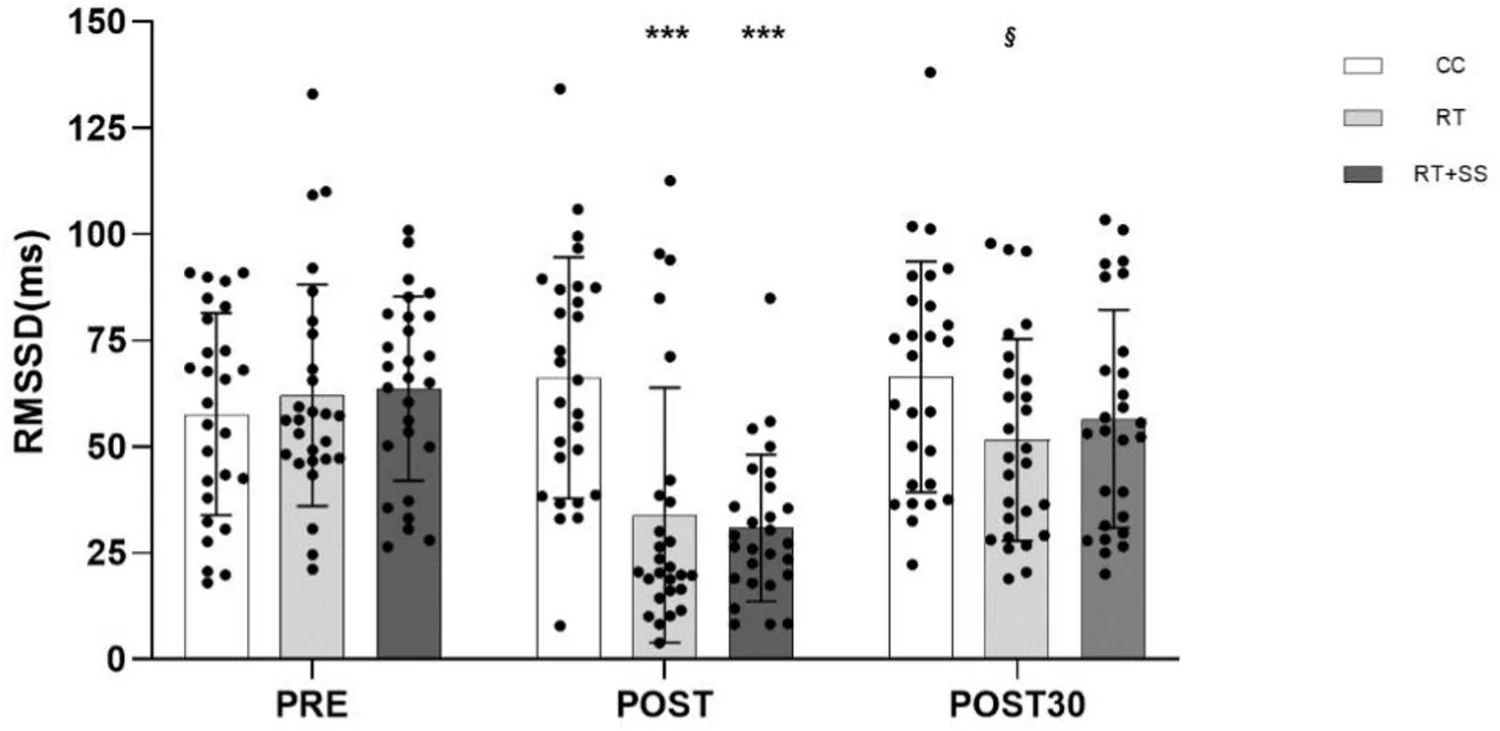
Root mean square of successive differences (RMSSD) measured at baseline (PRE), immediately after the intervention (POST), and 30 min post-intervention (POST30) during the control condition (CC), resistance training (RT), and resistance training followed by static stretching (RT + SS). Data are presented as mean ± SD with individual participant values. *p < 0.05, **p < 0.01, ***p < 0.001 for comparisons between POST and PRE within the same condition. **§**p < 0.05, **§§**p < 0.01, **§§§**p < 0.001 for comparisons between POST30 and PRE within the same condition

Finally, regarding time-domain indices, SDNN decreased significantly from BL to POST in both exercise conditions (RT: *p* < 0.001; d = 1.39; RT + SS: *p* < 0.001; d = 1.46), before returning to BL values at POST30. A similar trend was observed for RMSSD with the temporal patterns across conditions shown in Fig. 2. RMSSD decreased significantly from BL to POST in both exercise conditions (RT: *p* < 0.001; d = 1.29; RT + SS: *p* < 0.001; d = 1.29). However, at POST30, the RMSSD remained significantly different from BL only in the RT condition (*p* = 0.028; d = 0.57), while it returned to BL levels in RT + SS. No significant changes were observed in the CC.

### Hemodynamic changes

**Fig. 3:**
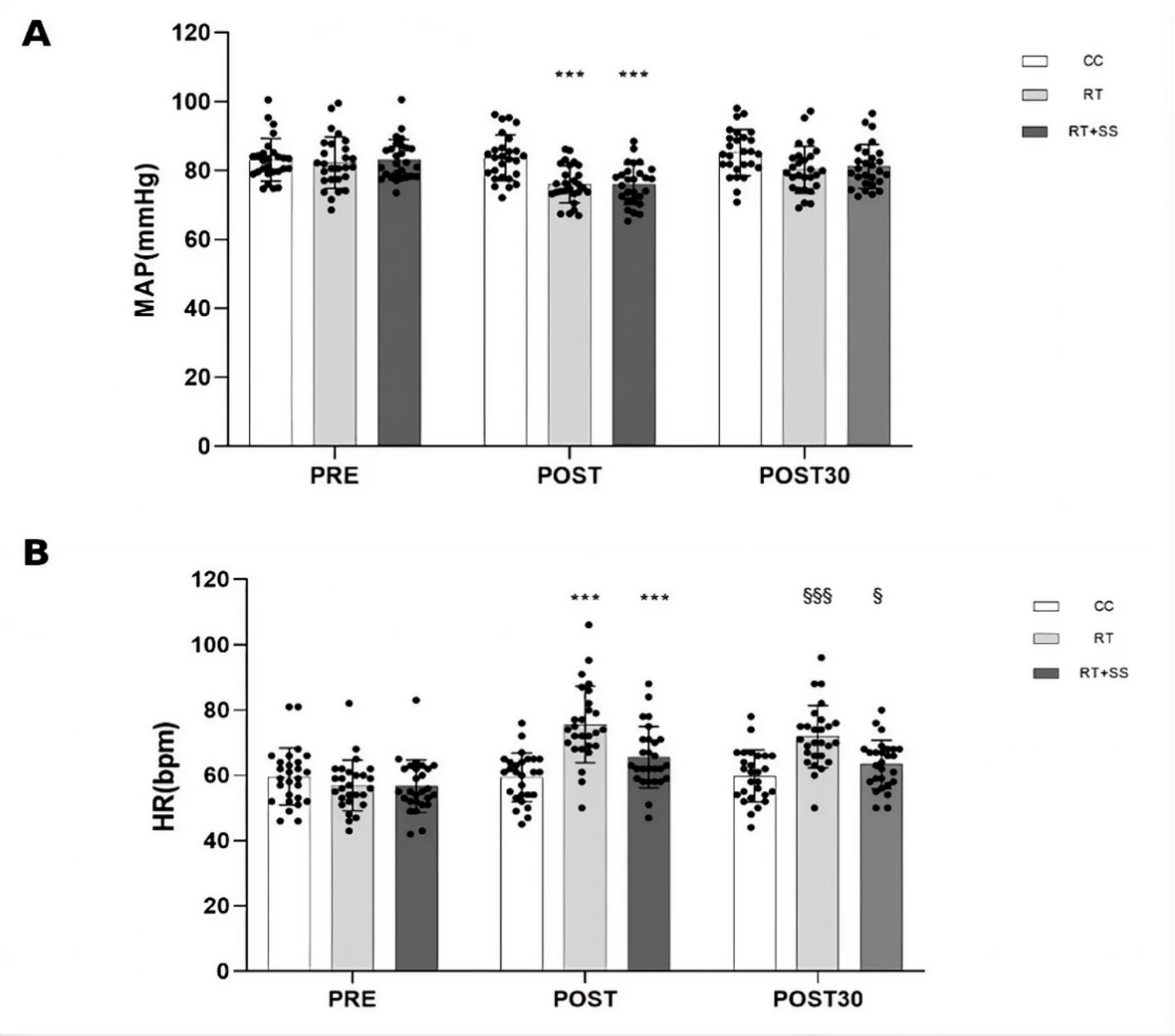
Mean arterial pressure (**a**) and Heart rate (**b**) measured at baseline (PRE), immediately after the intervention (POST), and 30 min post-intervention (POST30) during the control condition (CC), resistance training (RT), and resistance training followed by static stretching (RT + SS). Data are presented as mean ± SD with individual participant values. *p < 0.05, **p < 0.01, ***p < 0.001 for comparisons between POST and PRE within the same condition. **§**p < 0.05, **§§**p < 0.01, **§§§**p < 0.001 for comparisons between POST30 and PRE within the same condition

A significant condition × time interaction was observed for selected hemodynamic outcomes (*p* < 0.001). MAP decreased significantly from BL to POST in both exercise conditions (RT: *p* < 0.001; d = 0.98; RT + SS: *p* < 0.001; d = 1.10) and returned toward BL at POST30. HR significantly increased from BL to POST in both exercise conditions (RT: *p* < 0.001; d = 1.85; RT + SS: *p* < 0.001; d = 1.41) and remained significantly higher at POST30 in both conditions (RT: *p* < 0.001; d = 0.72; RT + SS: *p* < 0.05; d = 0.40). The temporal patterns across conditions for MAP and HR are shown in Fig. 3. No significant condition × time interaction was observed for SBP (*p* = 0.732, F = 0.49, ηp²=0.013). Conversely, DBP showed a significant condition × time interaction (*p* = 0.001, F = 16.0, ηp²=0.291), decreasing significantly from BL to POST following both RT and RT + SS and returning to BL at POST30 in both conditions. No significant changes were observed in the CC. The main RM-ANOVA outcomes for SBP and DBP are presented in Table 2.

**Table 2:** Synthesis of systolic and diastolic blood pressure for each condition and time point.

Analyzed variable	RT			RT + SS			CC			RM-ANOVA Time × condition interaction
	PRE	POST	POST30	PRE	POST	POST30	PRE	POST	POST30	
SBP (mmHg)	120.4 ± 2.7	120.3 ± 9.1	119.5 ± 7.5	120.8 ± 8.4	119.1 ± 9.1	120 ± 7.8	120.4 ± 8.9	120.2 ± 8.9	119.7 ± 9.3	p = 0.732; F = 0.49; np^2^=0.013
DBP (mmHg)	64.3 ± 7.1	54.2 ± 6.8*	59.5 ± 7.7*	64.2 ± 6.5	54.4 ± 6*	61.6 ± 7.3	64.3 ± 7.1	65.4 ± 6.7	67.8 ± 6.5	*p *< 0.001; F = 16.0; np^2^=0.291

Data are expressed as mean ± standard deviations

RT: Resistance training. SS: Static stretching. CC: Control condition PRE: Baseline Assessment. POST: Assessment after the end of the exercise session; POST30: Assessment 30 min after the end of exercise. SBP: Systolic blood pressure. DBP: Diastolic blood pressure. mmHg: millimeters of mercury. RM-ANOVA: Repeated Measures Analysis of Variance

*Significant difference compared to baseline (PRE).

## Discussion

This study evaluated whether adding SS after RT could influence acute early post-exercise recovery at the endothelial, autonomic, and hemodynamic levels. The results show that RT alone caused a significant reduction in FMD% at POST, with a tendency toward recovery at POST30, whereas this decrease was not observed when SS was performed after RT. Unlike FMD%, the BA diameter increased after both exercise conditions, but its recovery profile differed, remaining elevated at POST30 after RT alone and returning to BL after RT + SS. Both conditions also induced transient reductions in RMSSD and HF, associated with an increase in HR and a decrease in MAP. At POST30, RMSSD and HF remained below BL values following RT alone, whereas they no longer differed significantly from BL values following RT + SS. Overall, these results indicate that the addition of SS was associated with a different pattern of early vascular and autonomic recovery, while hemodynamic responses were essentially comparable between the two conditions. The transient reductions in FMD% and HRV indices observed following RT should not necessarily be interpreted as adverse physiological responses. In healthy, physically active young adults, these acute perturbations may represent predictable, time-dependent responses to exercise stress (Stanley et al. 2013; Dawson et al. 2013). From a vascular perspective, acute exercise influences blood flow, shear stress, blood pressure, and wall stress; repeated exposure to these hemodynamic stimuli may contribute to long-term functional and structural vascular adaptation (Green et al. 2017). Consequently, different recovery trajectories toward BL values observed in several autonomic and endothelial outcomes following RT + SS should be interpreted as a different short-term recovery trajectory rather than as evidence of a necessarily beneficial or clinically superior response. It remains to be determined whether this recovery strategy affects subsequent performance, chronic adaptation, or cardiovascular health. During RT alone, in line with current evidence (de Oliveira et al. 2020), we observed a marked reduction in FMD% with a tendency toward recovery that is incomplete at POST30, suggesting a transient impairment of endothelium-dependent vascular responsiveness that persists up to 30 min after the RT protocol. The scientific literature supports several physiological mechanisms that could help explain this pattern. Acute high-intensity RT may transiently reduce the bioavailability of NO and increase oxidative stress, potentially creating a vascular environment less supportive of endothelium-dependent vasodilation during early recovery (Dawson et al. 2013). Another possible mechanism potentially responsible for transient endothelial impairment may be an increased vasoconstrictive effect of the sympathetic nervous system, which might counteract endothelium-dependent vasodilation during the initial recovery phase (Atkinson et al. 2015). However, sympathetic activity was not directly assessed in the present study. In this context, the addition of SS may have altered the hemodynamic recovery profile, inducing changes in blood flow via the stretch-release mechanism; this may have generated a more favorable shear profile potentially including a reactive hyperemia, thereby leading to a faster recovery of endothelial responsiveness (Bisconti et al. 2020). Another possible hypothesis is that the stretch stimulus may increase Ca²⁺-dependent NO production through activation of nitric oxide synthase, representing an additional plausible pathway through which SS may have influenced endothelial recovery (Mueller et al. 2025; Zheng et al. 2016). Therefore, it would be plausible to view SS as a low-intensity stimulus, sufficiently significant to influence endothelial recovery. Our findings may align with the role of “vascular resiliency” attributed to SS (Caldwell et al. 2023), and with recent evidence showing that stretching performed after RT can improve the recovery of markers of vascular stiffness (Esaki et al. 2026). Autonomic responses may provide a complementary context for the various patterns of vascular recovery. In both conditions, the results show a marked reduction in RMSSD and HF due to the physiological stress caused by RT, leading to a transient reduction in cardiac vagal modulation; this finding suggests that, immediately after the procedure, the two protocols exhibited comparable profiles. This finding is consistent with the current literature, which documents an acute suppression of predominantly vagal HRV indices immediately after resistance exercise (Kingsley and Figueroa 2016; Marasingha-Arachchige et al. 2022). Nevertheless, our study observed significant differences in recovery profiles between the two conditions. Specifically, HF and RMSSD showed a more rapid return to BL levels at POST30 when SS was added to RT, suggesting that SS did not mitigate the acute stress induced by RT, but was associated with a different pattern in the early recovery phase. In this regard, previous evidence indicates that during the stretched phase there may be a temporary increase in sympathetic activity, while in the subsequent recovery phase there may be a more rapid rise in vagal markers, accompanied by increases in RMSSD and HF and a decrease in HR (Farinatti et al. 2011; Wong and Figueroa 2021). The mechanism behind this is not yet fully understood. The review by Wong and Figueroa (Wong and Figueroa 2021) highlights that during recovery, stretching training appears to lead to an increase in vagal modulation indices; however, the mechanisms are not clear and may include psychophysical relaxation responses, baroreflex sensitivity, and increased NO bioavailability. Furthermore, it is important to note that the same review highlights that in the acute phase the response differs between active and passive stretching, with the former being associated with greater sympathetic activation during performance, while the latter tends to favor the vagal component, although both converge toward marked vagal reactivation during recovery (Wong and Figueroa 2021). These autonomic responses could also influence vascular tone and thus provide a plausible mechanism linking the autonomic and endothelial recovery patterns observed following RT + SS. In our case, SS was shown to be associated with a faster early recovery of several HRV indices following RT. However, the parallel recovery of these indices does not directly prove that autonomic responses caused the vascular responses. Since baroreflex sensitivity, sympathetic activity, and mechanistic vascular variables were not directly assessed, these pathways remain hypothetical. The interpretation of LF and the LF/HF ratio must be distinct from that of RMSSD and HF. Although LF power and the LF/HF ratio have traditionally been associated with sympathetic modulation and sympathovagal balance, their physiological significance is more complex. LF power reflects the combined influences of the autonomic nervous system and the baroreflex, whereas the LF/HF ratio does not represent a direct quantitative measure of sympathovagal balance (Billman 2013; Reyes del Paso et al. 2013). Consequently, LF and the LF/HF ratio have been considered complementary outcomes, while the interpretation of autonomic recovery has been based primarily on the temporal responses of RMSSD and HF, which more specifically reflect cardiac vagal modulation (Laborde et al. 2017). Finally, regarding hemodynamic patterns, our study did not identify any significant differences between the two protocols, as MAP decreased at POST in both conditions and returned to BL values at POST30. These results are consistent with the current literature, which shows that acute hemodynamic responses following RT and SS can be variable and highly protocol-dependent, without necessarily indicating a clear systemic advantage of one condition over the other (Conceição et al. 2021; Costa e Silva et al. 2019; Benavides-Roca et al. 2025; Themistocleous et al. 2026). HR followed the same trend as MAP, although the direction of change differed, with a significant increase in both POST and POST30 following both conditions, indicating that the cardiovascular load from the exercise was still present during the recovery phase (Costa e Silva et al. 2019; Macedo et al. 2025). However, at POST30, in RT + SS compared to RT, appeard to be a trend toward favorable chronotropic recovery when SS was added at the end of the session (Thomas et al. 2021).

### Limitations

It is important to acknowledge some limitations. First, the study sample consisted of a homogeneous group of healthy physically active young men, which limits the generalizability of the results to broader or clinical populations. Second, this study examined only acute responses; therefore, it is not possible to make conclusions regarding chronic vascular or autonomic adaptations. Third, although the selected time points were appropriate for the established objective of comparing the immediate cardiovascular response and early recovery following RT with and without post-exercise SS, they did not allow for a detailed characterization of the entire recovery process. Therefore, transient responses observed between POST and POST30, as well as changes that persisted beyond 30 min, may not have been detected. Future studies involving more frequent or time-varying assessments could provide further insight into the kinetics of endothelial and autonomic recovery. Fourth, HRV provides an indirect assessment of cardiac autonomic modulation and does not allow for the independent quantification of sympathetic and parasympathetic activity. Therefore, findings related to the autonomic nervous system must be interpreted while acknowledging the physiological and methodological limitations inherent in HRV-derived indices. Fifth, the mechanistic variables that might help explain the observed vascular and autonomic responses, including shear stress, nitric oxide bioavailability, oxidative stress, sympathetic activity, and baroreflex sensitivity were not directly assessed. Therefore, the proposed physiological mechanisms should be considered plausible explanations rather than demonstrated mediators of the observed responses. Lastly, although FMD% was assessed using a standardized procedure, the absence of direct quantification of the shear stimulus limits the interpretation of whether the observed differences reflect intrinsic changes in endothelial reactivity or differences in the hemodynamic stimulus. Furthermore, FMD% is a derived variable calculated as the percentage difference between the baseline diameter and the maximum post-deflation diameter; therefore, its variability may be influenced by physiological fluctuations in arterial diameter and interindividual differences in vascular reactivity. This may partly explain the lower reproducibility commonly observed for FMD% compared to other measures.

## Conclusion

The addition of static stretching exercises at the end of a resistance training session was associated with a different early recovery pattern compared to resistance training alone, particularly in terms of autonomic and endothelial parameters. These findings suggest that static stretching may serve as a simple complementary modality to resistance training for modulating selected autonomic and endothelial responses during the first 30 min of recovery. Further studies are needed to clarify the physiological mechanisms underlying this phenomenon and to determine whether these potential acute effects can translate into clinically relevant chronic adaptations.

## Supplementary Information

Below is the link to the electronic supplementary material.

Supplementary Material 1

## Data Availability

The datasets generated and analyzed during the current study are available from the corresponding author upon reasonable request.

## Abbreviations

ANOVA: Analysis of variance
BA: Brachial-artery
BL: Baseline
CC: Control condition
DBP: Diastolic blood pressure
FMD: Flow-mediated dilation
HR: Heart rate
HRV: Heart rate variability
MAP: Mean arterial pressure
POST: Immediately after exercise
POST30: 30 min after exercise
RM-ANOVA: Repeated-measures analysis of variance
RT + SS: Resistance training+static stretching
RT: Resistance training
SBP: Systolic blood pressure
